# Combining Breath Figures with Mussel-Inspired Chemistry: An Easy Route to Finely Tunable Microporous Functional Surfaces

**DOI:** 10.1021/acsmaterialsau.5c00118

**Published:** 2025-11-05

**Authors:** Leonardo Moscolari, Gabriele Tullii, Adriano Vignali, Erika Kozma, Francesco Galeotti

**Affiliations:** † Istituto di Scienze e Tecnologie Chimiche “G. Natta” (SCITEC), 9327 Consiglio Nazionale delle Ricerche, via A. Corti 12, 20133 Milano, Italy; ‡ Center for Nano Science and Technology, 121451 Istituto Italiano di Tecnologia, Via Rubattino 81, 20134 Milano, Italy

**Keywords:** microporous films, polydopamine, plasmonic nanoparticles, multifunctional surfaces, SERS

## Abstract

Microporous surfaces are widely explored for their potential in applications such as sensing, catalysis, and photonics. However, achieving well-defined and reproducible porous architectures often requires complex or highly optimized fabrication techniques. In this work, we present a straightforward, ultrafast and scalable method for producing functional microporous films by combining spin-coated breath figures of cellulose acetate butyrate with polydopamine-assisted metallization. By systematically investigating the parameters influencing the breath figure process, we demonstrate precise control over the porosity and three-dimensional structure of the resulting films. The incorporation of polydopamine enables the subsequent formation of metal nanoparticles, imparting plasmonic and catalytic functionalities to the surfaces. This versatile platform offers new opportunities for the development of multifunctional materials tailored for advanced sensing and environmental applications.

## Graphical abstract

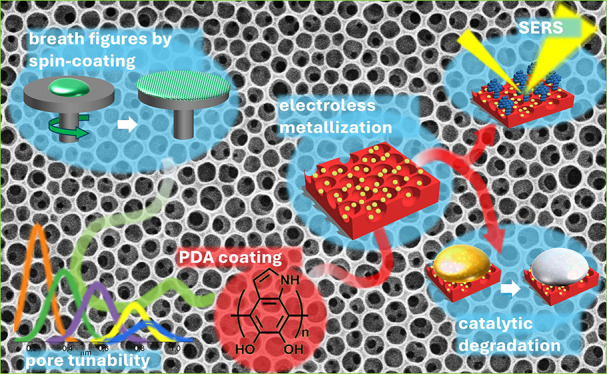

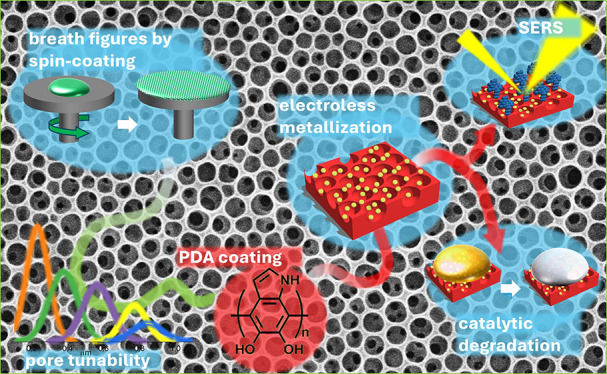

## Introduction

Microporous surfaces have been extensively studied for a long time because of their wide range of applications in fields such as catalysis, filtration, tissue engineering, antifouling, photonics and sensing.^1–5^ While creating microporous structures is not particularly challenging for nanofabrication scientists, the success of these applications relies on the ability to produce these structures in a reproducible manner using straightforward and efficient methods. Over the years, various techniques have been developed to create these surfaces, each with its own set of advantages and challenges.

One of the most straightforward and versatile methods for fabricating microporous surfaces is the breath figures (BFs) approach.^6,7^ This technique involves the formation of polymer films with tunable porous surfaces in a single fabrication step. The process typically entails drop casting a polymer solution onto a substrate, followed by evaporation under a flow of moistened gas. This triggers the solvent-evaporative cooling on the surface of the solution, where water vapor condenses and forms droplets at the interface between the solution and the air. These droplets then interact and arrange themselves into a hexagonal lattice. Once the solvent and water have completely evaporated, the remnants of the water droplets create pores in the polymer film. This results in a packed pore structure, which can be tailored by adjusting various parameters such as humidity, temperature, and polymer concentration. Compared to other suitable methods such as phase separation, reactive ion etching or lithography, which often require complex processing steps, specialized equipment, or harsh solvents, the BF process provides a simpler and more direct approach to fabricating microporous films, while microcontact printing can produce similar morphologies but lacks straightforward tunability. Despite its simplicity, the BF method often requires meticulous optimization to achieve the desired pore size, packing, and order.

To further streamline the fabrication process, a simpler method has been suggested: preparing BFs via spin-coating.^8,9^ This technique involves rapidly spinning the substrate to evenly distribute the polymer solution under controlled humidity, which can improve control over pore formation and minimize the need for extensive optimization. While spin-coating may not produce the highly ordered patterns achievable with dropcasting BFs, it offers advantages in terms of reproducibility and implementation speed.

Pushed by the idea of rendering the fabrication as simple as possible and within the reach of any laboratory, we propose a method for creating microporous functional surfaces by combining spin-coated BFs based on cellulose acetate butyrate (CAB) and polydopamine (PDA).

CAB was chosen as porous scaffold material for several compelling reasons. It is a low cost chemically modified natural polymer of wide use in automotive and industrial coatings, packaging, laminates and protective coverings, in cosmetics and pharmaceutic industry.^10^ The controlled acetylation and butyration of natural cellulose make it soluble in a variety of organic solvents, which facilitates the spin-coating process.^11^ The degree of substitution in CAB can be adjusted, allowing for the fine-tuning of its physical and chemical properties. Its good film-forming properties ensure the creation of uniform and stable films. Moreover, CAB exhibits favorable mechanical properties, such as flexibility and toughness, which are essential for maintaining the integrity of the porous structures during and after fabrication.

To date, only a limited number of studies have explored in depth the patterning of CAB by BF approach. Park and Kim, who were the first to propose it, investigated the impact of water content at two different spinning speeds. They also suggested that these patterns could be used as antireflection coatings.^12^ Dário et al. investigated how the molecular weight of CAB and the acetone content in the solvent mixture affect the spin-coating of BFs under controlled relative humidity.^13^ Blachechen et al. studied the effect of relative humidity during spin-coating process on the structural characteristics of CAB films.^14^ In another paper targeted to the development of light diffusers for OLEDs, Lim and Suh qualitatively evaluated pore density and shape of CAB BFs prepared at three CAB concentrations and three different spinning rates, under high humidity condition generated by an ultrasonic humidifier.^15^ Nevertheless, none of these studies considered the comprehensive set of parameters necessary for effective process management, which was the primary focus of the current study.

Moreover, despite the high versatility and tunability of the so obtained microporous surfaces, so far CAB BFs have been proposed just as antireflection coatings and light diffusers in OLEDs.^12,15^ Our next goal was to expand the potential uses of CAB microporous films by incorporating plasmonic features, with polydopamine playing a crucial role in this achievement.

Mussel-inspired polydopamine coating is in fact a valuable tool for surface modification due to its the strong adhesive properties and its ability to form a thin, uniform coating on porous surfaces.^16,17^ This coating can then serve as a versatile platform for the in situ formation of various metal nanoparticles, which can impart additional functionalities such as catalytic activity or plasmonic amplification of Raman signals in sensing applications.^18–23^

Here we developed a method to decorate the surface of CAB microporous films with metal nanoparticles using a straightforward approach involving polydopamine functionalization and electroless metallization. This paves the way for plasmonic effect-related applications such as Surface Enhanced Raman Scattering (SERS) substrates in sensing devices and catalytic degradation of organic pollutants.

## Materials and Methods

### Preparation of CAB Films

Cellulose acetate butyrate (average Mn ∼ 12,000) was purchased from Merck. CAB was dissolved in tetrahydrofuran (THF) at a concentration of 50 and 100 mg/mL. In both cases 5% (v/v) Milli-Q water was added. Water additions of 1, 2.5, 7.5, and 10% (v/v) were investigated for the first CAB concentration. To prepare the porous films 50 μL of CAB mixture was deposited on a clean round coverslip (Ø = 12 mm) and rotated by spin coater. Variations of different spinning parameters were tested: revolutions per minute (rpm, between 100 and 5000), acceleration (ACL, between 5 and 50), and rotating time (between 1 and 10 s). Typically, the process is completed within 4 s and the morphology of the film does not change for longer rotating time. The emergence of an opaque, slightly iridescent surface–reminiscent of mother-of-pearl–indicates the successful formation of the BF pattern. All the spinning process was conducted under ambient conditions. Flat samples were prepared by spin-coating the dry THF CAB solution (50 g/L) at 3000 rpm into a humidity-free glovebox.

### Polydopamine Coating

Dopamine hydrochloride was purchased from Fluorochem and tris­(hydroxymethyl) aminomethane (tris) was purchased from Merck. In a Petri dish (Ø = 90 mm), 80 mg of dopamine hydrochloride was dissolved in 40 mL of 25 mM tris solution (pH = 10.5). To avoid delamination from the border due to surface tension, the CAB samples were protected with an external poly­(dimethylsiloxane) ring. Furthermore, just before immersion, a drop of ethanol was placed on the porous samples to increase hydrophilicity and immediately immersed in the dopamine solution for 2 h at room temperature. Magnetic stirring was maintained using a 12 mm stirring bar rotating at 800 rpm to prevent the formation of a PDA film on the surface of the reaction solution and uncontrolled deposition on the samples. Then, the samples were rinsed with water and incubated again in fresh dopamine solution for another 2 h. Finally, PDA-coated samples were rinsed with water and dried at room temperature.

### Gold Nanoparticle In Situ Growth

Tetrachloroauric acid (HAuCl_4_) was purchased from Merck. The PDA-coated samples were placed on the bottom of a 50 mL crystallizer in a 0.1 mg/mL water solution of HAuCl_4_. The reaction solution was stirred gently for 16 h. Samples were rinsed with water and let dry at room temperature. The final gold content on each kind of substrate was determined by thermogravimetric analysis (TGA), carried out on a PerkinElmer TGA 7 instrument at 10 °C/min in the thermal range from 50 to 750 °C under nitrogen flow. Once 750 °C was reached, the temperature was held constant for 10 min under a dry air atmosphere to erase carbonaceous residue.

### Morphological and Optical Characterization

Scanning electron microscopy (SEM) analysis was performed by using a Phenom Pro Desktop scanning electron microscope (Thermo Fisher Scientific Inc., Eindhoven, The Netherlands), at an accelerating voltage of 15.0 kV, after sputtering the samples with 5 nm of Au. AFM analysis was performed by using a NT-MDT NTEGRA (Limerick, Ireland) instrument. The detailed procedure used for assessing the pose size and distribution is reported in the Supporting Information. Optical properties were evaluated using a PerkinElmer Lambda 900 UV/Vis/NIR Spectrometer.

### Evaluation of SERS Performances

SERS measurements were performed with a commercial Raman microscope (Renishaw InVia) with a 785 nm laser source, equipped with a 50× objective. Prior to measurements, the system was calibrated using a silicon standard. The substrates were incubated into a biphenyl-4-thiol (4-BPT, Sigma-Aldrich, 97%) solution in methanol for 1 h, washed with pure methanol to remove the excess unbound molecules, and left to dry in air. Both flat and microporous CAB-PDA-AuNPs substrates were tested. The following spin-coating conditions, leading to an average porosity of 770 nm, were used for the preparation of the microporous substrates: CAB concentration = 50 g/L, water content = 7.5%, spinning rate = 3000 rpm, acceleration = 50. Flat samples were prepared by spin-coating the dry THF CAB solution (50 g/L) at 3000 rpm into a humidity-free glovebox. Each SERS spectrum was acquired by performing 100 accumulations with an integration time of 1 s and a laser power on the sample of about 0.17 mW. The obtained spectra were background-corrected with polynomial fit to the second degree. Origin 2018 was employed for data analysis.

### Catalytic Degradation Tests

The catalytic degradation of methyl orange (MO) was studied by mixing 1.0 mL of a 3.05 mM MO solution with 0.2 mL of a freshly prepared 0.846 M NaBH_4_ solution, resulting in final concentrations of 2.5 mM MO and 140 mM NaBH_4_. A 100 μL aliquot of this mixture was deposited onto the catalytic surface. Both flat and microporous CAB-PDA-AuNPs substrates were tested. For the preparation of the microporous substrates, the same spin-coating conditions previously described for SERS, were used. Subsequently, 10 μL aliquots were taken at different time intervals, transferred into plastic cuvettes, and diluted with 500 μL of deionized water to record the corresponding UV–Vis absorption spectra. The changes in dye concentration over time were monitored using a PerkinElmer UV/Vis/NIR Lambda 900 spectrometer.

## Results and Discussion

### Fabrication and Characterization of CAB-Based Microporous Surfaces

BF formation relies on the condensation or micrometric water droplets on the evaporating polymer solution. This water is typically introduced into the system either by directing a stream of moist air or nitrogen over the polymer solution or by controlling the system’s relative humidity (static procedure), with typical effective values ranging from 65 to 90%.^24^ These approaches are effective for dropcasting BFs, leading to highly ordered arrays of closely packed cavities on the surface of the pure polymer or even hybrid film.^25,26^ However, the straightforward nature of spin-coating as a processing technique led us to optimize a BF method involving spin-coating instead of dropcasting, aiming to make it faster and more reproducible. One drawback is that controlling the humidity within the spin-coating system is complicated and poses reproducibility issues. To address this problem, we added small amounts of water directly to the CAB solution and performed spin-coating without additional humidity control. The use of a ternary system is possible thanks to the complementarity of the different materials involved: THF, which was selected as the main solvent; CAB, which is soluble in THF and serves as the constituting material of the porous films; and water, which is partially soluble in THF and a poor solvent for CAB, triggering pore formation. The photographs and wide-field SEM images presented in the Supporting Information (Figure S1) demonstrate that our method achieves complete coverage of the 12 mm coverslip substrate with a highly homogeneous BF pattern.

The amount of water dissolved in the CAB THF solution is crucial for pore formation, determining both their packing and their size. The other parameters that we considered for tuning the porosity of CAB BFs are polymer concentration, spinning rate and acceleration.

Initially, we tested the BF formation by adding water to THF in different amounts. For these experiments, CAB concentration was kept at 50 g/L, the spinning rate was set at 3000 rpm and the acceleration parameter (ACL) was 50. The results are summarized in Figure 1. As shown by the SEM views, the average pore size increases with the amount of added water, ranging from 0.23 to 0.84 μm of diameter going from 1 to 10% (v/v), respectively.

**Figure 1:**
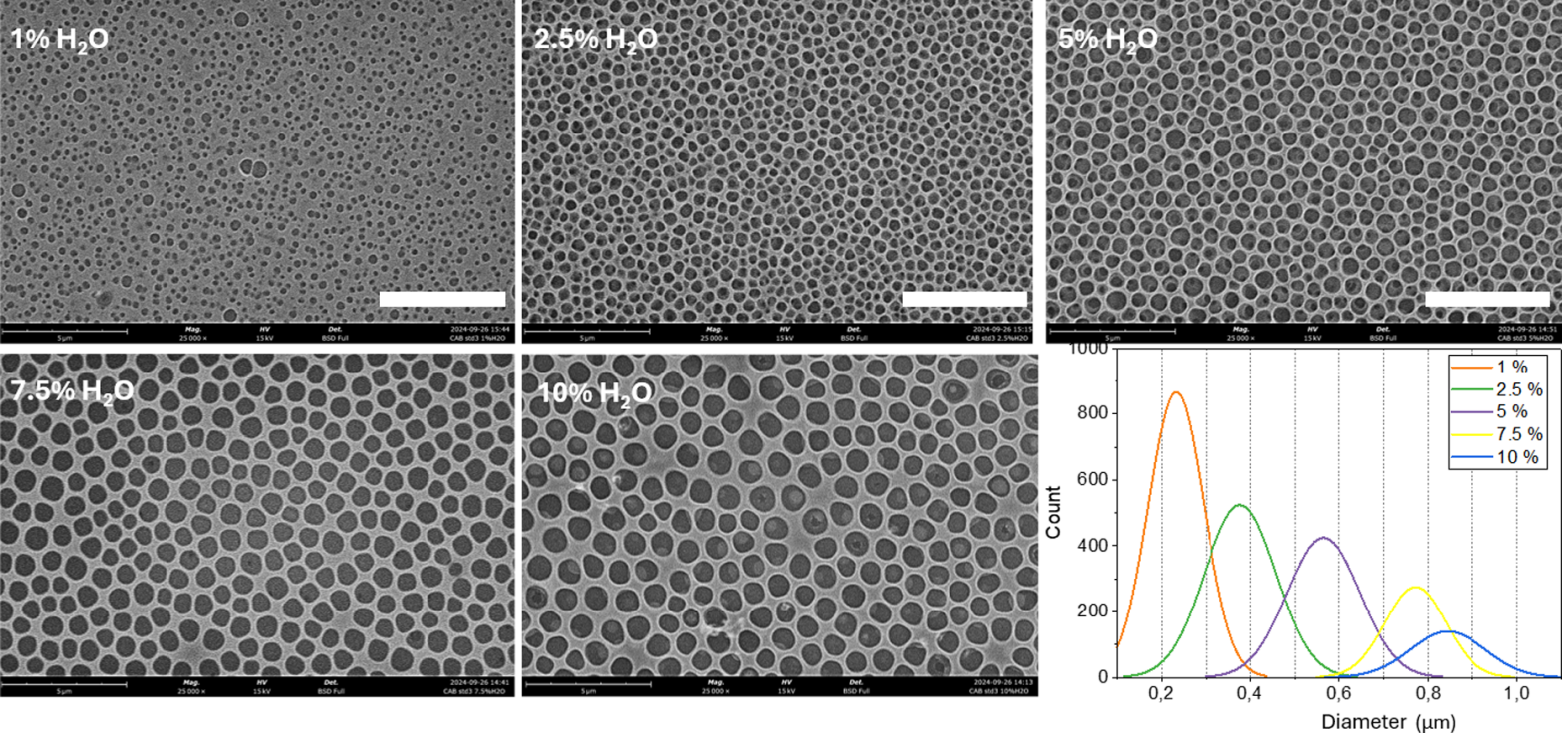
SEM micrographs of CAB films prepared by varying the amount of dissolved water (reported on the images) and corresponding distribution plots of pore diameters. The remaining parameters are kept constant for all samples: concentration = 50 g/L, spinning rate = 3000 rpm, acceleration = 50. Size bars are 5 μm.

We set the 1% as the minimum water content, because in these conditions the porosity starts to be evident, even if the small cavities are not closely packed. Water content below 1% led to flat CAB films with a few cavities randomly distributed, indicating that the natural humidity of the laboratory (between 25 and 40%) is not sufficient for obtaining complete BF arrays by spin-coating. To obtain completely flat CAB films, on the other hand, a free humidity ambient (glovebox) and dry THF were needed.

The effect of water content on the cavity packing is clearly evident by looking at Figure 1. By increasing the water content from 1 to 2.5 and 5%, the cavities grow in size and become closely packed. At 7.5% the further size increment leads to a diminishing of the packing, which becomes more evident at 10%. Given these results, the water content of 5% turned out to be optimal for obtaining closely packed BFs of average size, and was selected as fixed parameter for the following experiments.

The spinning rate determines the speed of the film formation process and hence of BF formation, as well as the thickness. Obviously, both the parameters have an impact on the porosity of BF arrays, as illustrated in Figure 2. At spinning rates between 1000 and 5000 rpm, homogeneous and closely packed cavities are formed. The average pore size is inversely proportional to the spinning rate, making it an effective tool for tuning pore diameter. The representative images, shown in Figure 2, show that the largest pores, approximately 0.9 μm, are produced at 1000 rpm, while at 5000 rpm, the pores are reduced to 0.42 μm.

**Figure 2:**
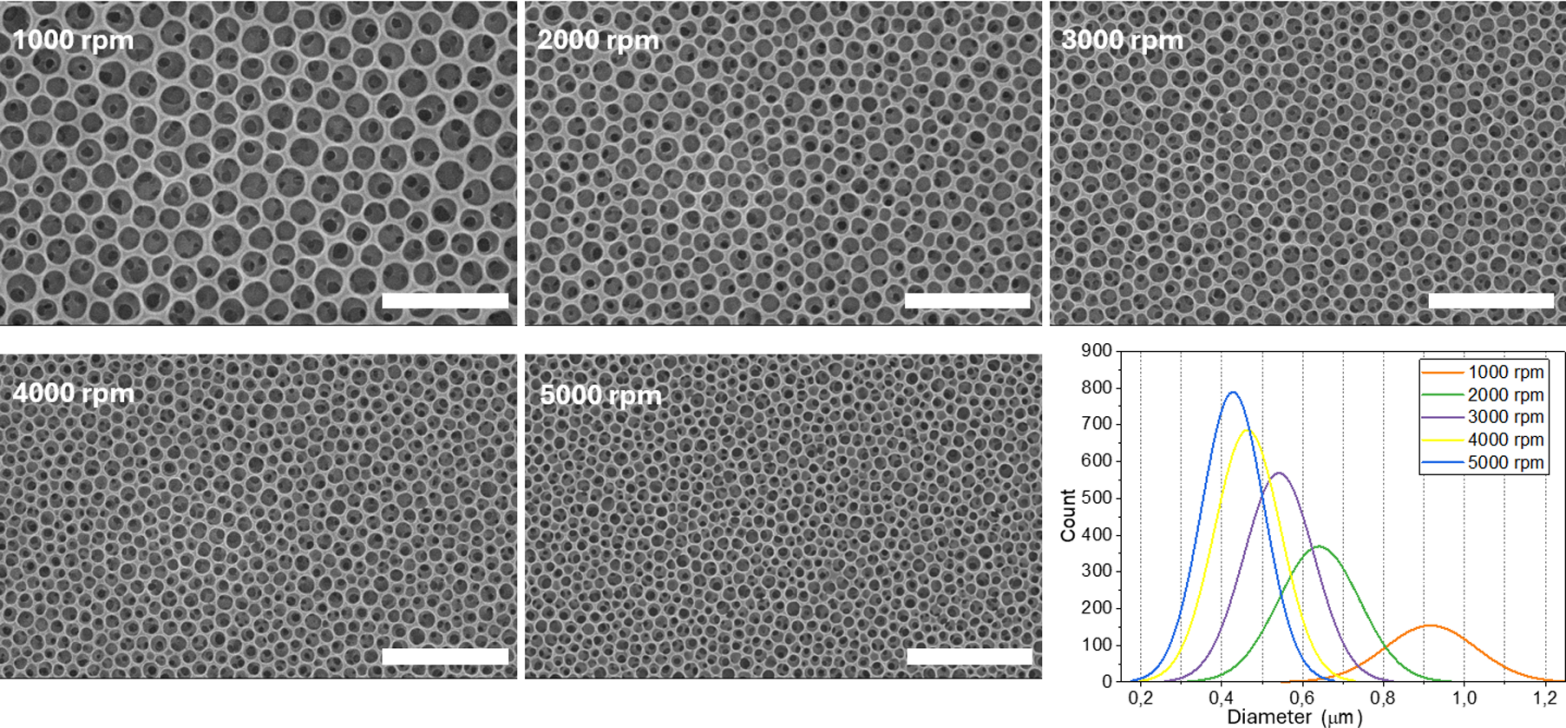
SEM micrographs of CAB films prepared by varying the spinning rate (reported on the images) and corresponding distribution plots of pore diameters. The remaining parameters are kept constant for all samples: water content 5%, concentration = 50 g/L, acceleration = 50. Size bars are 5 μm.

In addition to the rate, also the spinning acceleration (ACL) is relevant for BF formation, since this parameter influences the centrifugal force acting on the CAB solution during the initial stage of the process. Optimizing ACL is particularly important for viscous solutions, as gradually increasing the rotation speed ensures even distribution of the material across the substrate. Even if the viscosity of CAB solutions in THF is not so high, ACL impacts the BF process during spin-coating; the first few seconds of rotation are vital for the formation of water droplets on the developing film, which in turn affects its morphology. By keeping the target rotation speed to 3000 rpm, we produced BF films setting different ACL values, from 5 to 50. In the spin-coating system that we used for this study, these values correspond to rotational acceleration of 665 and 6650 rpm/s, respectively. The complete list of ACL values and time needed to reach the target rotation speed is reported in Table 1.

**Table 1:** Changing Parameters for Spin-Coating of CAB

ACL	spinning acceleration (rpm/s)	acceleration time (s)	target speed (rpm)
5	665	4.5	3000
10	1330	2.3	3000
15	1995	1.5	3000
25	3325	0.9	3000
50	6650	0.5	3000

As shown in Figure 3, increasing the ACL initially reduces the average pore size until it reaches a plateau. The porosity decreases from 0.85 μm at ACL = 5, to 0.70 μm at ACL = 10, and approximately 0.55 μm at ACL = 15. Further increasing the ACL does not reduce the cavity diameter, indicating that the target rotation speed is quickly reached and becomes the dominant factor.

**Figure 3:**
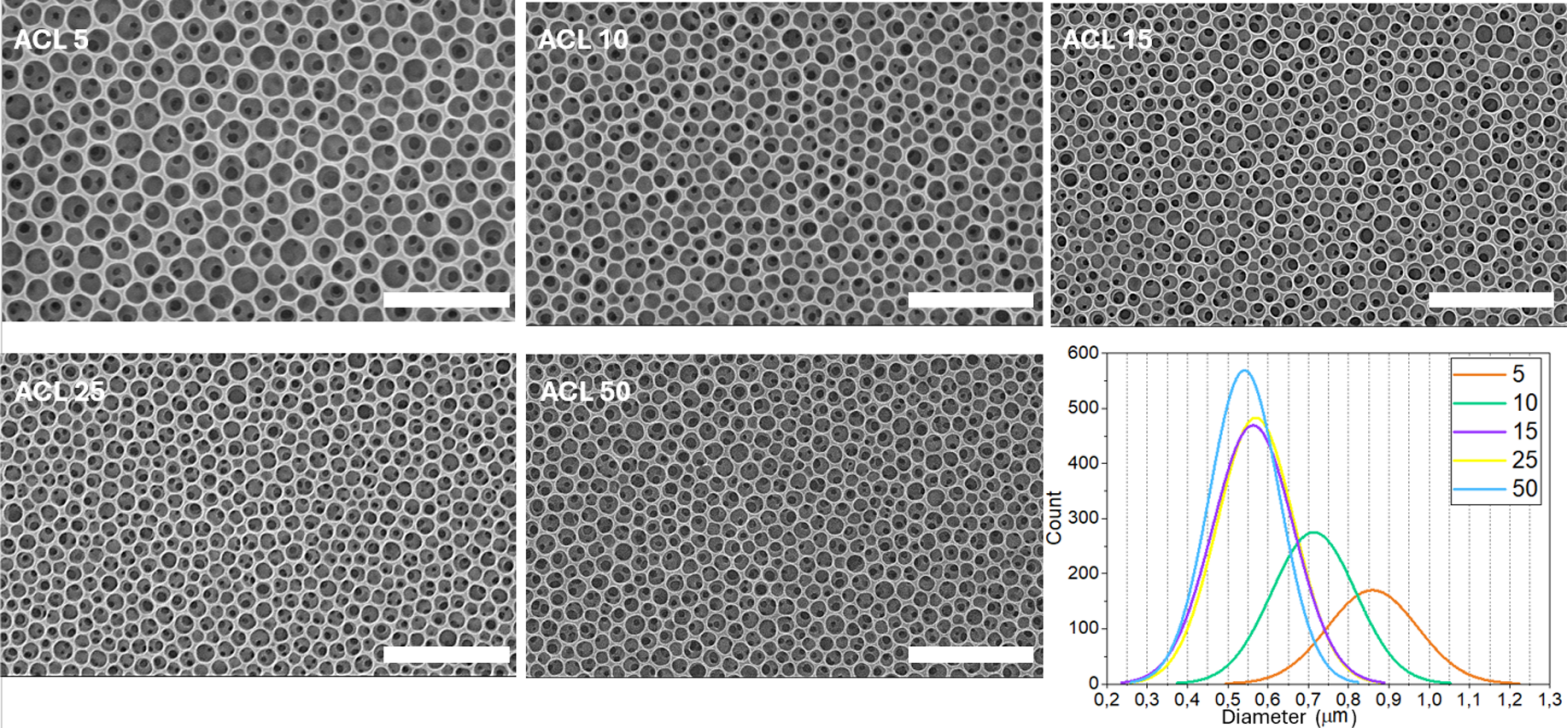
SEM micrographs of CAB films prepared by varying the acceleration parameter (ACL, reported on the images) and corresponding distribution plots of pore diameters. The remaining parameters are kept constant for all samples: spinning rate = 3000 rpm, water content 5%, concentration = 50 g/L. Size bars are 5 μm.

It is also worth noting that the final pore size is determined within the very first seconds of the process, and this effective time decreases as the ACL increases. By stopping the spin-coating at different times, we verified that the final pore size is reached after only 2 s at ACL 50, while it takes 4 s at ACL 5.

CAB concentration is another useful parameter for tuning the porosity of CAB BF films. In the BF technique, increasing the polymer concentration typically leads to the formation of larger pores, due to several interrelated factors.^27–29^ First, higher polymer concentrations result in a more viscous solution. This increased viscosity slows down the evaporation rate of the solvent, allowing more time for water droplets to condense and grow larger on the polymer surface. Additionally, a greater amount of polymer material is available to form the matrix around the water droplets, which helps stabilize larger droplets and ultimately results in larger pores. Overall, the combination of slower evaporation, larger initial droplet size, and a more robust polymer network contributes to the formation of larger pores when the polymer concentration is increased.

As shown in Figure 4, doubling the CAB concentration of the spin-coated solution nearly doubled the mean pore size, increasing from 0.58 to 0.95 μm. This confirms the general rule for BF formation. Moreover, the SEM images of the film’s lateral section reveal that even the three-dimensional arrangement of the BF array is influenced by the concentration. At 50 g/L, the concentration produces mono or bilayers of cavities, whereas increasing the CAB concentration to 100 g/L results in multiple layers with cavities arranged on 3–4 different levels.

**Figure 4:**
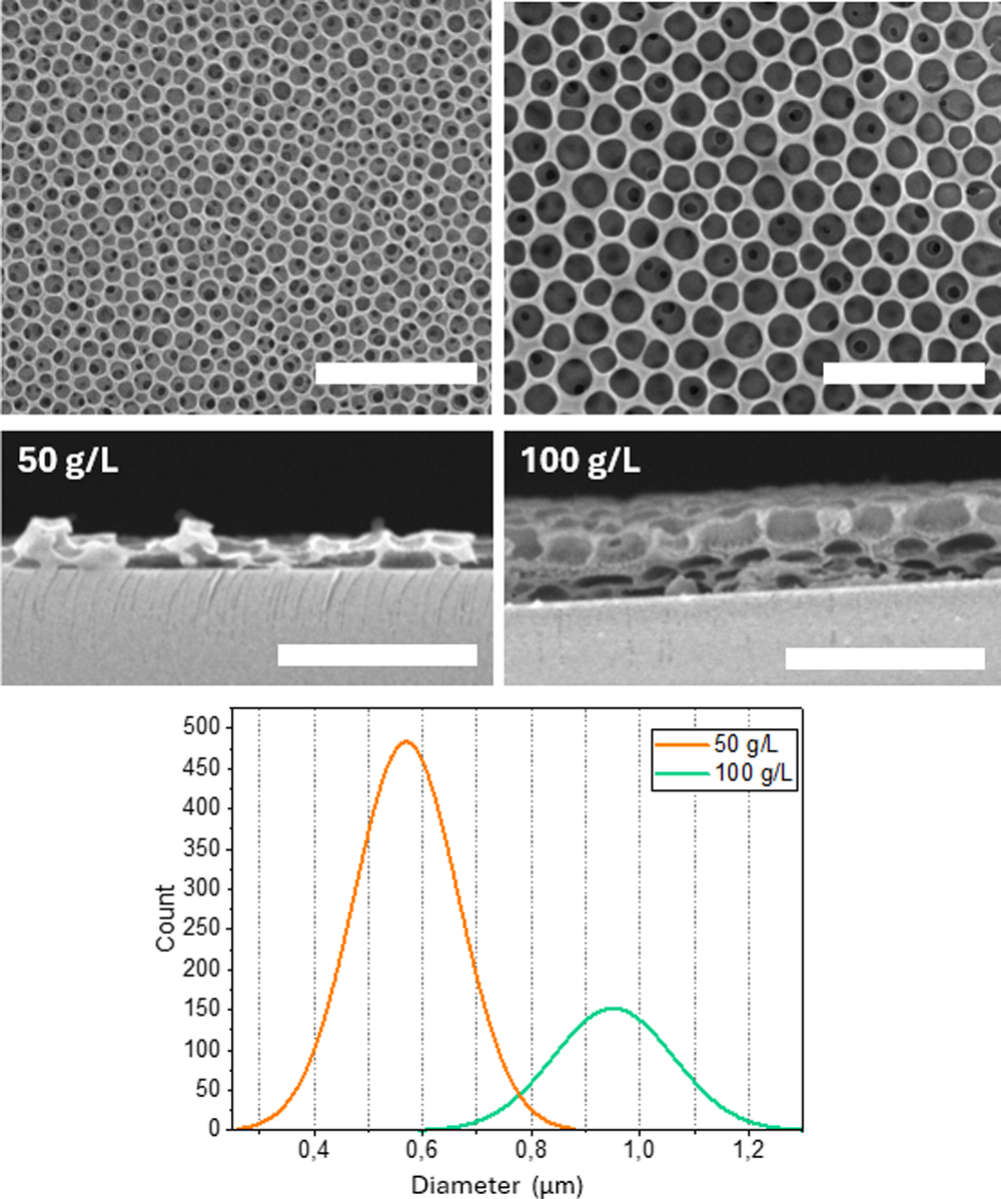
SEM micrographs of CAB films prepared at two different concentrations (50 and 100 g/L) and corresponding distribution plots of pore diameters. The remaining parameters are kept constant: spinning rate = 3000 rpm, water content 5%, acceleration = 50. Size bars are 5 μm.

The impact of the investigated spin-coating parameters on CAB film porosity is summarized in Table 2.

**Table 2:** Average Porosity Values under the Various Operational Conditions Investigated

CAB conc. (g/L)	dissolved H_2_O (%)	spinning rate (rpm)	ACL	average pore diameter (μm)
100	5.0	3000	50	0.95
50	1.0	3000	50	0.23
50	2.5	3000	50	0.37
50	5.0	3000	50	0.55
50	7.5	3000	50	0.78
50	10.0	3000	50	0.84
50	5.0	1000	50	0.91
50	5.0	2000	50	0.64
50	5.0	4000	50	0.47
50	5.0	5000	50	0.42
50	5.0	3000	5	0.85
50	5.0	3000	10	0.72
50	5.0	3000	15	0.56
50	5.0	3000	25	0.55

### Polydopamine Functionalization and Decoration with Plasmonic Nanoparticles

To expand the potential uses of CAB microporous films, including the possibility of incorporating plasmonic features, we functionalized them with a thin layer of PDA. This step was achieved by incubating the CAB membranes in a dopamine solution in the presence of tris buffer, for 4 h. This process generates a layer of about 20 nm (evaluated by measuring the thickness of a PDA layer grown on a piece of Si wafer, in the same conditions), which is suitable for preserving the original morphology of the BF array and is thick enough for enabling the further step of metal NP in situ formation. However, our first attempts to functionalize the microporous films with PDA did not lead to a conformal coating. As shown in Figure 5a, the PDA film formed mostly over the cavities of CAB, sealing them, instead of following the porous morphology. We ascribed this result to the scarce tendency of dopamine solution to enter inside the air-filled cavities. To address this issue, we introduced a preconditioning step for the CAB film using ethanol, which was expected to increase the wettability of the porous film. An additional challenge arose from the prolonged incubation (4 h) with dopamine hydrochloride, which led to the substantial formation of PDA particles in solution—evidenced by a dark brown coloration—and their subsequent adhesion to the film surface. To address this, we implemented a two-step incubation protocol: the film was first incubated for 2 h, then washed and reincubated in a fresh dopamine solution for another 2 h. This strategy effectively reduced the number of adhered PDA particles. As shown in Figure 5b, ethanol pretreatment followed by two sequential 2-h polymerization steps resulted in a conformal PDA coating that preserved the original BF array structure, with minimal particle attachment.

**Figure 5:**
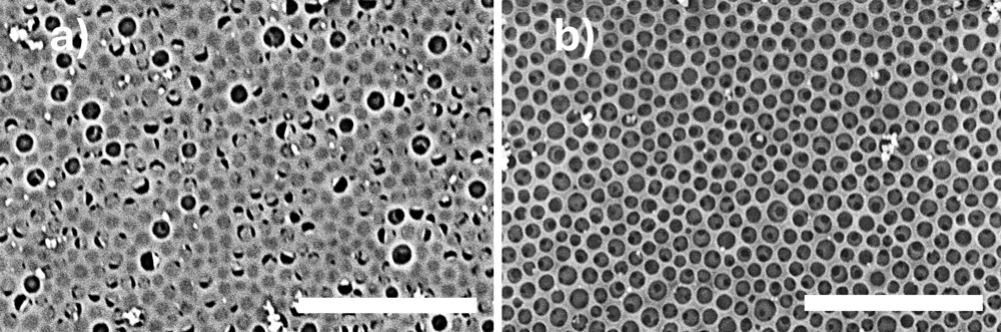
SEM micrographs of PDA-coated porous film obtained without (a) and after (b) ethanol conditioning step. Size bars are 5 μm.

After coating with PDA, the CAB films turn brownish, indicating that autopolymerization of dopamine has occurred, forming colored unsaturated chemical structures (Figure S2).^30,31^

Incorporating metal nanoparticles into a film or substrate through in situ growth is a straightforward method for introducing plasmonic properties into the system. These properties can be employed to create functional surfaces for applications such as sensing, photocatalysis, photothermal therapy, and photoacoustic imaging, among others.^32–34^ CAB BF arrays, featuring regular and tunable surface porosity along with an enhanced surface area, are promising candidates. The outer PDA layer enriches the porous films with catechol groups, whose oxidation to quinones facilitates the in situ reduction of metal ions, leading to the formation of metal nanoparticles.^35^ By exploiting the chemical reducing ability of PDA, we decorated the obtained microporous surfaces with gold nanoparticles, thereby endowing them with plasmonic properties. After 16 h of incubation with a diluted HAuCl_4_ solution (0.1 mg/mL), the resulting films exhibit a progressive darkening accompanied by a bluish hue, as depicted in Figure S2.

In Figure 6a, a SEM magnified view of a PDA-coated CAB porous film obtained after Au nanoparticles in situ growth is shown. Both the external and the internal surface of the cavities are decorated with nanoparticles, appearing as tiny bright spots. The few bigger spots (hundreds of nm large) randomly distributed on the film are ascribable to PDA particles, also coated by Au. The plasmonic effect introduced by the Au nanoparticles is evidenced by the UV–Vis absorption spectra shown in Figure 6b: the films covered with nanoparticles exhibit an absorption band around 550 nm, which corresponds to the plasmonic resonance of Au nanoparticles of 40–60 nm.^36^ A second band, observed around 450 nm and also present in the porous CAB film without metal nanoparticles, can be attributed to the quasi-periodic morphology imparted by the BFs.

**Figure 6:**
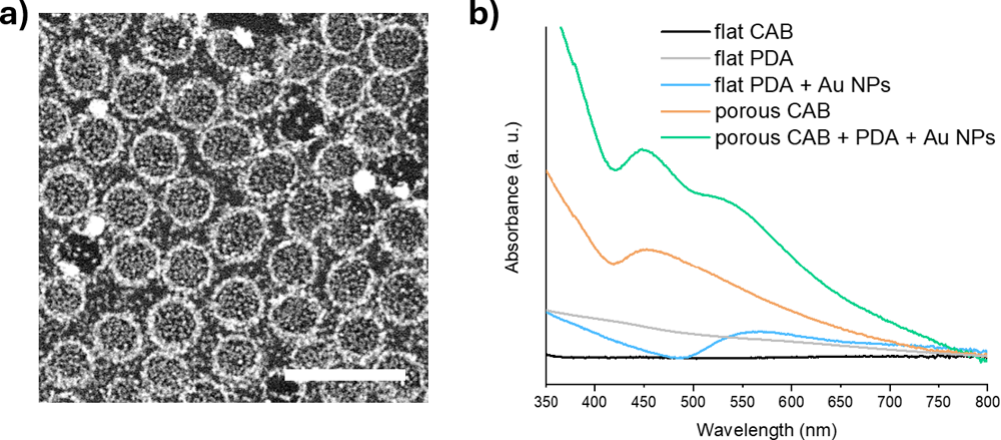
(a) SEM view of polydopamine-coated breath figure array after in situ growth of Au nanoparticles. Scale bar is 2 μm. (b) UV–Vis absorption spectra of flat and porous films before and after polydopamine coating and Au nanoparticles in situ growth.

### Application in Sensing and Degradation of Organic Pollutants

The functionalization of microporous CAB films with plasmonic nanoparticles renders them suitable as substrates for SERS-based sensing platforms. This suitability arises from several synergistic factors: the localized enhancement of the electromagnetic field due to the formation of plasmonic hot spots, the analyte preconcentration effect facilitated by the presence of PDA, and the increased surface area provided by the BF array. Collectively, these features are expected to significantly amplify the Raman signal of analyte molecules adsorbed on the film surface. To investigate this potential, we measured the Raman response of 4-BPT, a standard Raman reporter molecule, adsorbed onto the plasmonic CAB films. The 4-BPT molecules self-assemble on gold forming semicovalent bonds, leading to a uniform monolayer on the gold surface.^37^ Figure 7 shows the Raman spectra of 4-BPT absorbed from a 1.0 mM solution onto flat and microporous plasmonic substrates based on CAB-PDA. Both substrates are effective in amplifying the intensity of Raman peaks compared to bare PDA layer on glass, which lacks the Au nanoparticles layer and does not lead to the detection of 4-BPT Raman signal at the operating conditions under use.

**Figure 7:**
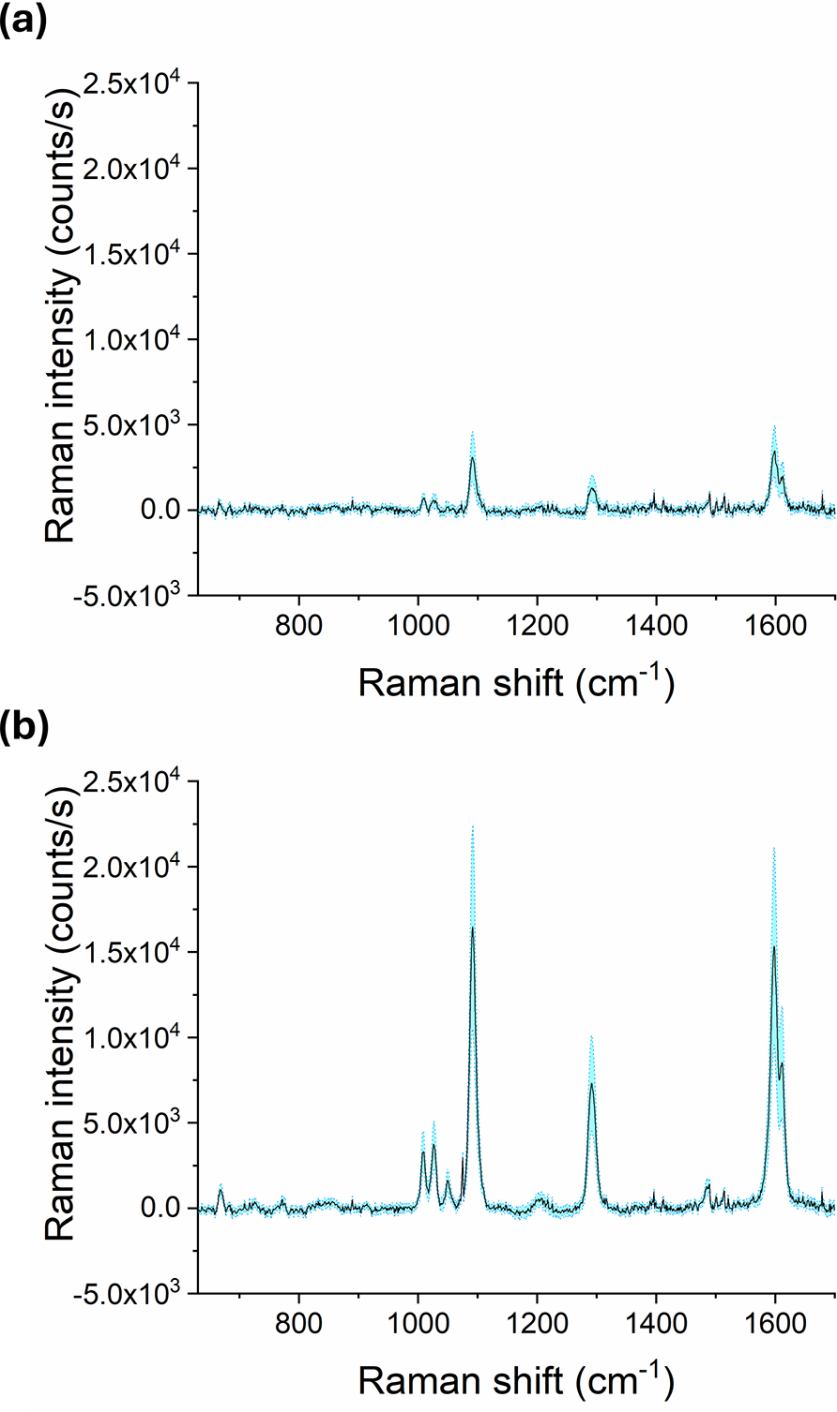
Average Raman spectra (solid lines) of 4-BPT absorbed from a 1.0 mM solution onto flat (a) and microporous (b) plasmonic substrates based on CAB-PDA. The spectra were obtained by averaging the signal obtained from 40 different regions, belonging to 4 different samples, for each condition. Cyan shaded areas represent the standard deviation.

By focusing on the vibrational mode located at 1590 cm^–1^ (associated with the aromatic C=C stretching of the biphenyl ring and commonly used as a reference due to its intensity and spectral clarity) we find that the microporous substrate enhances the Raman signal by approximately five times as compared to the flat substrate. This enhancement is attributed to both the increased surface area and the photonic effects associated with the BF morphology. Thermogravimetric analysis revealed that the microporous films contain approximately three times more gold than the flat ones (results reported in Figure S4). This implies that, during Raman measurements, the laser spot on the microporous films interacts with a substantially greater amount of AuNPs. Therefore, the increased surface area of the microporous structure is likely a dominant contributing factor.

The estimated enhancement factor (EF) for the microporous film was 2.2 × 10^4^, in line with self-assembled plasmonic structures, while still offering room for optimization (see Supporting Information for the full EF calculation).^22,38^ The structural integrity and sensing reliability of the substrates were validated through repeated analyte detection on the same microporous sample and by the sustained presence of AuNPs following multiple sensing treatments (Figures S5 and S6). Furthermore, the possibility to finely tune the substrate’s porosity enables the design of tailored cavities based on the size of the target analyte—ranging from molecules, to globular proteins, to bacteria—allowing them to be surrounded by plasmonic hot spots and thereby maximizing detection sensitivity.^39^ In this context, the strong adhesive properties of PDA, which allow it to anchor to a wide variety of materials and surfaces, are expected to play a pivotal role. This strategy will be further explored and optimized in a forthcoming study.

To further investigate the practical applications of our microporous functional surfaces, we evaluated their catalytic activity in degrading hazardous organic dyes. As a proof-of-concept, we selected methyl orange (MO), a widely used azo dye in industries such as textiles, paper, and cosmetics, which poses significant environmental challenges due to its chemical stability and resistance to natural degradation. When discharged into water bodies, it can cause toxic effects on aquatic life, disrupt ecosystems, and contribute to visual pollution.

Gold nanoparticles are well-established catalysts for the degradation of organic dyes, particularly in the presence of a reducing agent like sodium borohydride (NaBH_4_). This catalytic activity is largely attributed to the surface plasmon resonance effect of Au nanoparticles, which enhances their ability to mediate electron transfer processes.^40^ In this context, the reduction of MO involves a redox mechanism, where NaBH_4_ acts as the electron donor, MO acts as the electron acceptor and Au nanocrystals serve as a conductive bridge, by adsorbing both MO and BH_4_^–^ ions, bringing them in close proximity and facilitating the transfer of electrons from the donor to the acceptor.

Typically, catalytic nanoparticles are dispersed directly in the solution containing the dye. However, in our study, we aimed to test the catalytic performance of immobilized Au nanoparticles on microporous surfaces. This approach not only simplifies catalyst recovery but also demonstrates the potential of our structured films as solid-state catalytic platforms.

In aqueous solution, MO exhibits an orange color and shows a characteristic absorption peak at 468 nm (Figure 8a). Upon the addition of NaBH_4_, the solution remains relatively stable for several minutes, with the absorption peak intensity decreasing by only 10% after 30 min. This indicates that, in the absence of a catalyst, the reduction of MO by NaBH_4_ proceeds very slowly, and approximately 90% of the dye remains unreacted. However, when the MO + NaBH_4_ solution is brought into contact with the plasmonic CAB-PDA-Au surface, the reduction rate increases significantly, suggesting that the Au nanoparticles enhance the dye’s reduction (Figure 8b,c). The intensity of the 468 nm peak decreases by 91% and 95% in the presence of the flat and porous plasmonic surfaces, respectively. Notably, most of the MO reduction occurs within the first 3 min of incubation, during which the absorption peak decreases by 70 and 78% for the flat and porous surfaces, respectively. The slightly higher degradation efficiency observed with the porous surface (Figure 8c), compared to the flat one (Figure 8b), can be attributed to its increased surface area. This enhanced surface promotes better contact and interaction with the MO + NaBH_4_ solution, further supporting the potential of our approach for developing solid-state catalytic surfaces.

**Figure 8:**
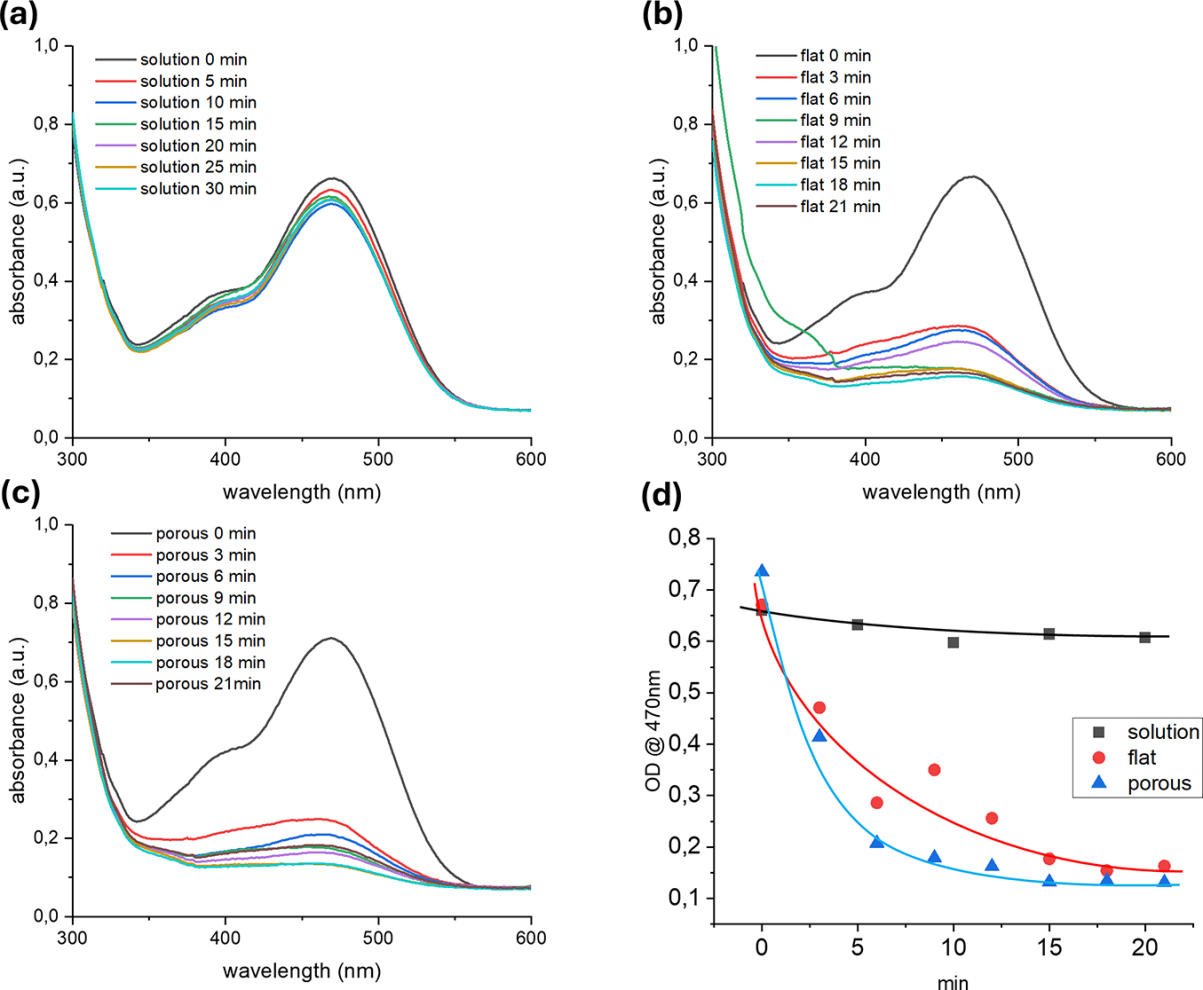
Catalytic degradation of methyl orange dye monitored via UV–Vis absorption spectra over time after NaBH_4_ addition: (a) without any catalyst; (b) with flat CAB-PDA-Au substrate; (c) with porous CAB-PDA-Au substrate, and variation of the optical density at 470 nm in the three experiments over time (d).

## Conclusions

In this study, we presented a simple and accessible method for fabricating microporous functional surfaces by combining spin-coated BFs of CAB with PDA-assisted metallization. Building upon the inherent advantages of CAB—its film-forming ability, mechanical robustness, and solvent compatibility—we optimized the BF process to produce reproducible porous architectures under controlled conditions. Through a comprehensive investigation of the key parameters influencing the BF process, we achieved precise control over the porosity and three-dimensional architecture of the CAB films. This level of tunability is essential for tailoring the surface morphology to specific functional requirements. The proposed ultrafast procedure takes between 2 and 4 s only. The subsequent PDA coating enabled the in situ formation of metal nanoparticles on the porous scaffold, imparting plasmonic functionality to the films in a straightforward and scalable manner. The fabrication of microporous and PDA-coated surfaces in a single step was also explored, pushing further the simplicity of the method.

This approach not only simplifies the fabrication of microporous surfaces but also broadens their potential applications beyond traditional uses such as antireflection coatings and light diffusers. The resulting metal-decorated CAB films hold promise for use in SERS sensing and catalytic degradation of organic pollutants. In fact, we found that the microporous surfaces decorated with Au nanoparticles enhance the Raman signal of a target molecule (4-BPT) by a factor of 5 compared to flat surfaces, with an estimated enhancement factor EF of 2.2 × 10^4^, indicating their strong potential as SERS substrates. Moreover, we demonstrated as a proof-of-concept that our CAB–PDA–Au films can catalyze the degradation of a water-persistent organic dye (methyl orange), reducing its concentration to about 10% of the initial value within 18 min.

Overall, our method offers a versatile and scalable platform for the development of multifunctional porous materials, paving the way for future innovations in sensing, catalysis, and beyond.

## Supplementary Material



## Notes

Large SEM view of microporous CAB film; photographs of films after each fabrication step; pore size calculation procedure; TGA plots; repeatability of SERS measurements; stability of AuNP decoration after sensing; SERS enhancement factor estimation procedure (PDF)
