# Supplementary material for: Combining Breath Figures with Mussel-Inspired Chemistry: An Easy Route to Finely Tunable Microporous Functional Surfaces
Source: ACS Mater Au. 2025 Nov 5;6(1):154–62. doi: 10.1021/acsmaterialsau.5c00118 (PMC12810034; doi:10.1021/acsmaterialsau.5c00118)
Supplement: Supplementary file 1 [file mg5c00118_si_001.pdf]

Supporting Information

Combining Breath Figures with Mussel-Inspired Chemistry: An Easy Route to Finely Tunable Microporous Functional Surfaces

*Leonardo Moscolari,[1] Gabriele Tullii,[2] Adriano Vignali,[1] Erika Kozma,[1] and Francesco Galeotti *,[1]*

[1] Istituto di Scienze e Tecnologie Chimiche "G. Natta" (SCITEC), Consiglio Nazionale delle Ricerche, via A. Corti 12, 20133 Milano, Italy.

[2] Center for Nano Science and Technology, Istituto Italiano di Tecnologia, Via Rubattino 81, 20134 Milano, Italy.

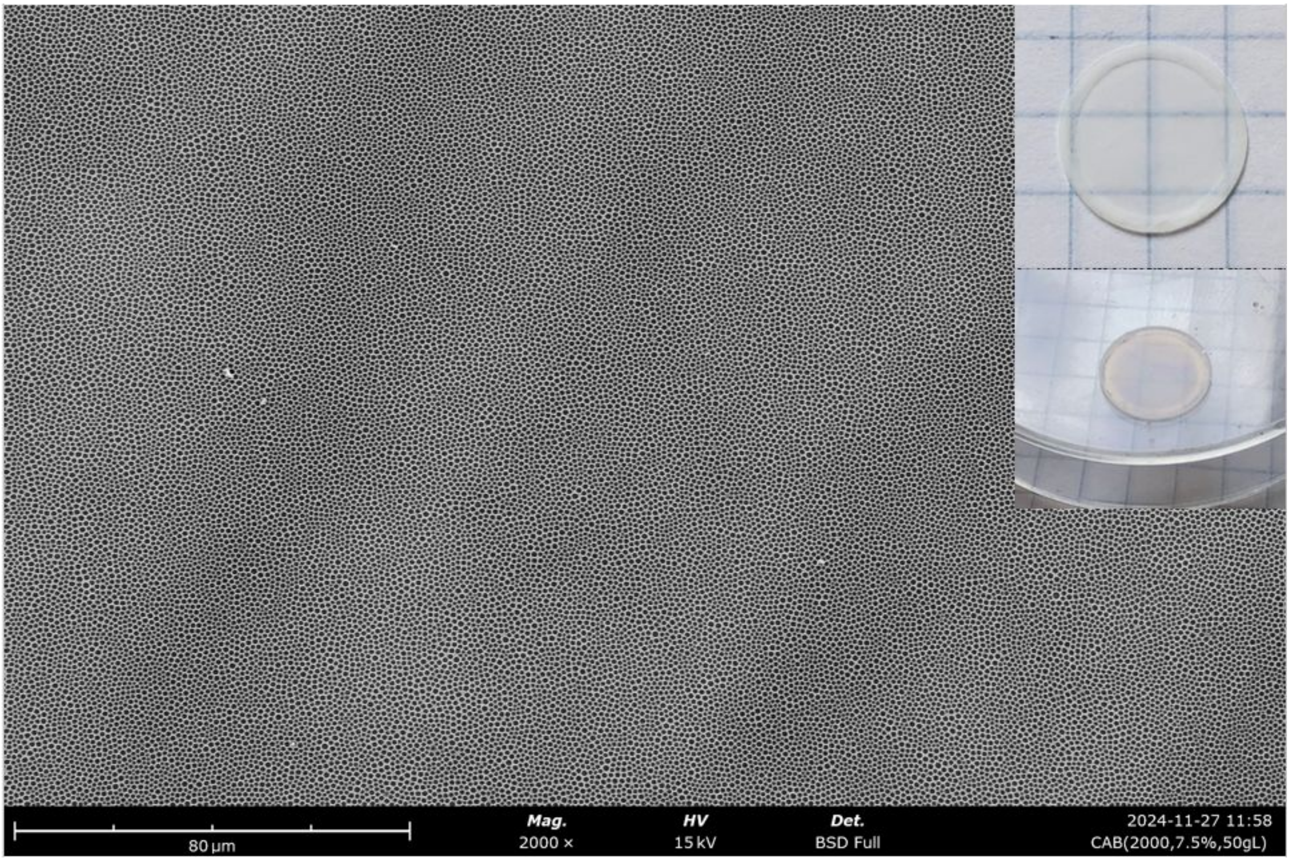

Figure S1. SEM micrography of CAB BF array showing the homogeneity of the porous structure over a wide area. In the insets: representative photograph of a CAB porous film on glass coverslip in orthogonal (top) and tilted (bottom) view.

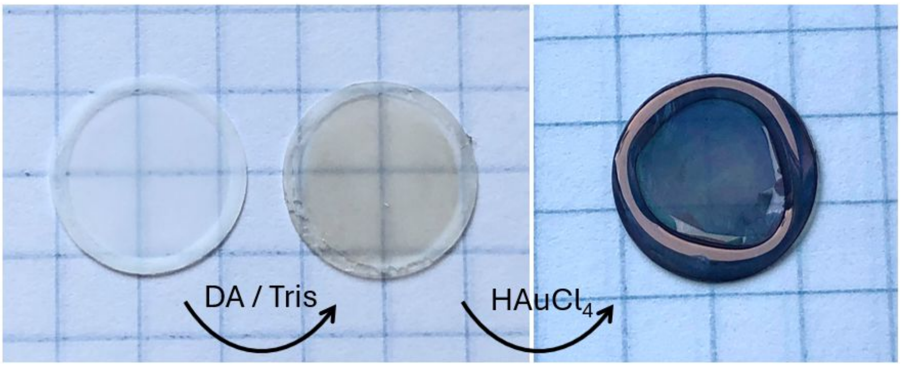

Figure S2. Photographs of porous CAB film (left), after coating with PDA (middle) and after decoration with Au nanoparticles (right).

Pore size calculation procedure

Pore dimensions were measured using ImageJ software.[1] A SEM image was first imported into ImageJ (**Figure S3-A**), and the scale was set. The image was then converted to binary format via "*Process*" → *"Binary"* → *"Make Binary"*, resulting in a black-and-white image (**Figure S3-B**). To ensure identification of the pores, the binary image was inverted using "*Image*" → *"Adjust"* → *"Threshold"*. In most cases, selecting "*Apply*" is enough, however, minor manual adjustments to the threshold levels were occasionally required to enhance contrast and isolate pores effectively (**Figure S3-C**). Subsequently, internal pore regions were refined using the "*Process*" → *"Binary"* → *"Fill Holes"* command (**Figure S3-D**). Pore size analysis was conducted by selecting "*Analyze*" → "*Analyze Particles*". During this step, parameters for size and circularity were adjusted to filter out artifacts. Furthermore, the options "*Show: Outlines*", "*Display Results*", and "*Exclude on Edges*" were selected to visualize the analyzed pores (**Figure S3-E**), obtain numerical data, and exclude partially visible pores at the image boundaries, respectively. For each fabrication condition, we measured four different SEM images. The resulting area values were used to calculate the pore diameter distribution under each condition.

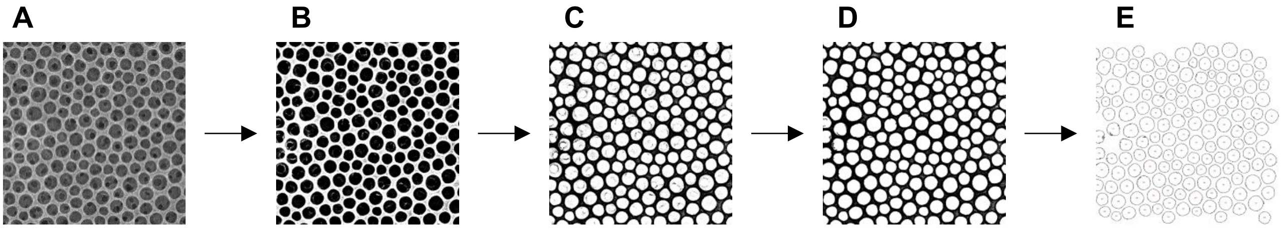

Figure S3. A) Original SEM image. B) Binary image obtained using the "Make Binary" command. C) Inverted binary image following adjustment and application of "threshold" function. D) Binary image with refined internal pore regions after "Fill Holes" command. E) Final output displaying pore outlines generated via the "Analyze Particles" function, highlighting the pores selected for measurement.

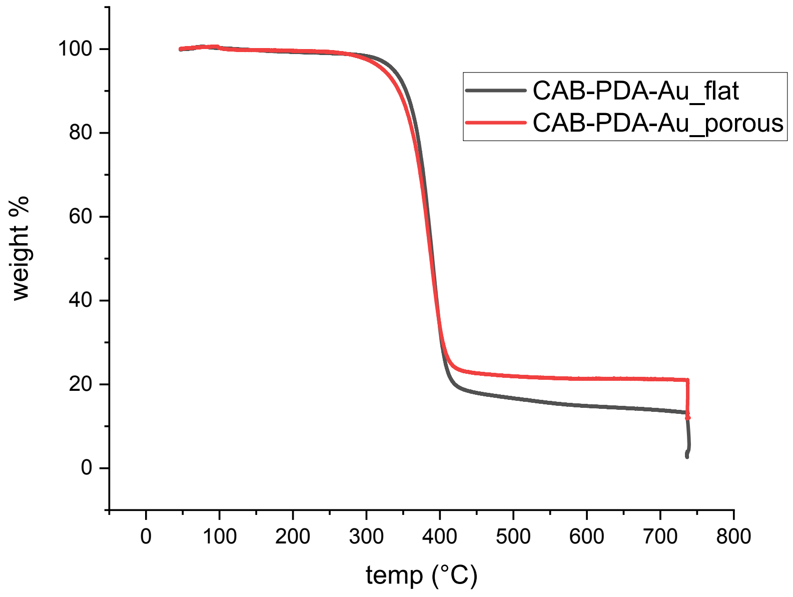

Figure S4. TGA curves of flat and microporous PDA-coated CAB films decorated with AuNPs. Both samples exhibit approximately 80-90% weight loss beginning at 300 °C, attributed to the thermal degradation of the organic components (CAB and PDA). A further 10% of mass (carbonaceous residue) is lost after air inlet at 750 °C. The residual mass corresponds to the AuNP content: 2.7% for the flat film and 11.9% for the microporous film. When normalized to the projected area of the film (i.e., the area of the glass substrate), this corresponds to 5.3×10^{-5} mg/mm² of Au for the flat film and 1.6×10^{-4} mg/mm² for the microporous one, leading to a ratio of ~1:3.

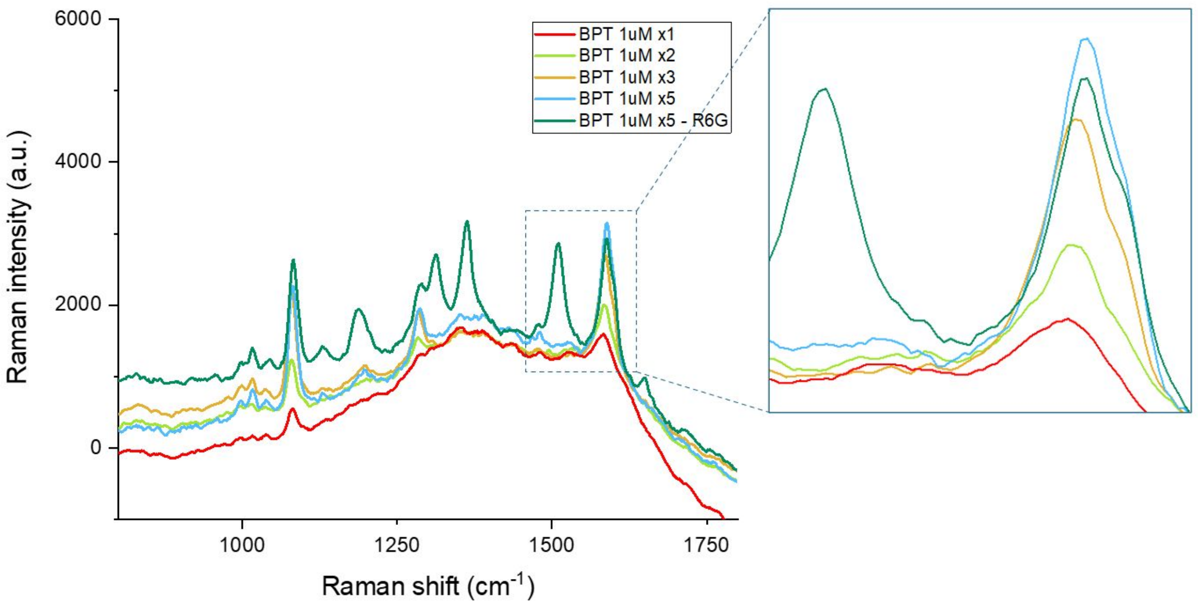

Figure S5. Raman spectra of a microporous plasmonic substrate based on CAB-PDA after repeated 1-hour incubations in a 1.0 μM solution of 4-BPT. The progressive increase in the intensity of the1590 cm^{-1} peak after each incubation is highlighted in the inset. Following the fifth treatment with 4-BPT, the same substrate was incubated in a 100 μM solution of rhodamine 6G (dark green trace), resulting in additional peaks at 1313, 1362, and 1510 cm^{-1}.

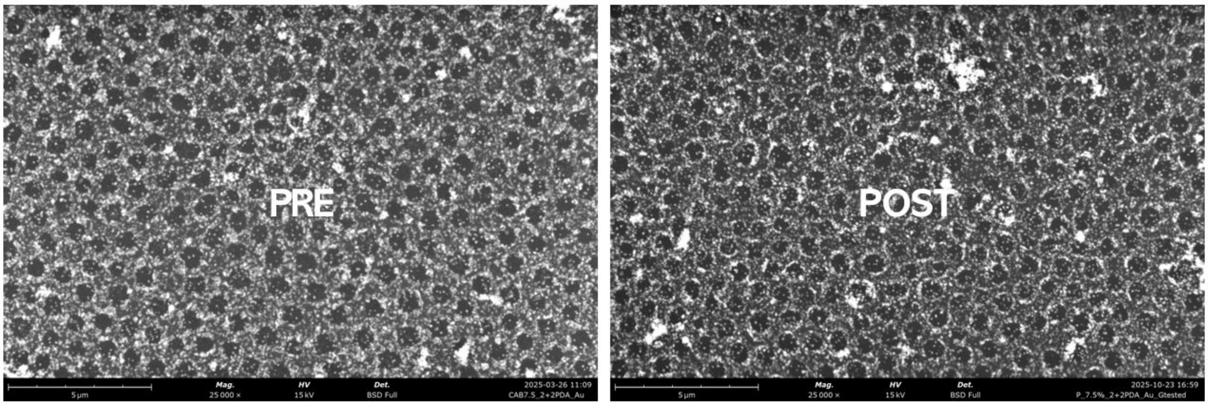

Figure S6. SEM view of a porous CAB-PDA-AuNP film before and after multiple incubation with 4-BPT and Raman analysis, showing that the AuNP decoration remains intact after sensing.

SERS enhancement factor estimation

To evaluate the enhancement factor, the SERS substrates were incubated into a 1 mM solution of biphenyl-4-thiol (4-BPT) in methanol for 1 h, then washed out in pure methanol, in order to remove the excess of free molecules, and left to dry in the air. In this way, a self-assembled monolayer of BPT is formed on GNP surface and used for the SERS measurements. For recording the Raman spectrum of 4-BPT, a small amount of powder was placed on a microscopy slide.

For estimating the EF, the following equation was used:

$$EF = \left(\frac{I_{SERS}}{I_{RS}}\right)\left(\frac{N_{RS}}{N_{SERS}}\right)$$

where I_{SERS} and I_{RS} are the intensities of the band at 1590 cm^{-1} for the SERS and Raman spectra of 4-BPT, respectively, N_{RS} is the average number of the BPT molecules in the scattering volume for the Raman measurements and N_{SERS} is the number of molecules in the laser scattering area probed during SERS measurements [2-4].

N_{RS} was calculated using the follow equation:

$$N_{rs} = A_{las} h \rho$$

where A_{las} is the area of the focal spot of the laser, h is the height of the volume of the 4-BPT powder contributing to the Raman signal and ρ is the density of 4-BPT expressed in number of molecules per nm^3 (3.44). We determined h for the measurement configuration by transferring a silicon wafer across the focal plane of the objective lens and recording the intensity of the Raman signal at 520 cm^{-1} from the silicon. By integrating the intensity of the Raman signal over distance and dividing the calculated value by the largest observed Raman signal we obtained h = 37.6 µm

N_{SERS} was calculated according to the equation:

$$N_{SERS} = A_{LAS}\frac{R}{\sigma}$$

where A_{las} is the area of the focal spot of the laser, σ is the surface area occupied by the immobilized molecule considering a footprint of 1 nm^2 for 4-BPT,[5] and R is the roughness factor, calculated as:

$$R = \frac{\Gamma A_{sphere}}{A_{pro}}$$

where Γ is the surface coverage of the nanoparticle estimated from the SEM analysis, A_{sphere} is the surface area of the nanoparticles, and A_{pro} is the projected surface area of the nanoparticles on the glass surfaces (the shape of the nanoparticles was assumed to be an ideal spherical form).

Full calculations:

$$\text{EF} = \left(\frac{I_{SERS}}{I_{RS}}\right)\left(\frac{N_{RS}}{N_{SERS}}\right) = \left(\frac{I_{SERS}}{I_{RS}}\right)\left(\frac{A_{las}h\rho}{\frac{A_{las}R}{\sigma}}\right) = \left(\frac{I_{SERS}}{I_{RS}}\right)\left(\frac{h\rho}{\frac{\Gamma A_{sphere}}{A_{pro}\sigma}}\right) = \left(\frac{I_{SERS}}{I_{RS}}\right)\left(\frac{h\rho}{\frac{\Gamma 4\pi r^2}{\pi r^2 \sigma}}\right) = \left(\frac{I_{SERS}}{I_{RS}}\right)\left(\frac{h\rho}{4\frac{\Gamma}{\sigma}}\right)$$

Measured values with our setup:

h = 37.6 μm = 3.76×10^4 nm

ρ = 3.44 mol/nm^3

Γ = 0.7 (coverage 70%)

σ = 1 nm^2/mol

$\frac{I_{SERS}}{I_{RS}}$ = 7.7 (intensity of the peak at 1590 cm^{-1})

Calculated EF= 2.2×10^4

(1) Schneider, C. A.; Rasband, W. S.; Eliceiri, K. W. NIH Image to ImageJ: 25 years of image analysis. *Nature Methods* **2012**, *9* (7), 671-675. DOI: 10.1038/nmeth.2089.

(2) Smythe, E. J.; Dickey, M. D.; Bao, J.; Whitesides, G. M.; Capasso, F. Optical Antenna Arrays on a Fiber Facet for in Situ Surface-Enhanced Raman Scattering Detection. *Nano Letters* **2009**, *9* (3), 1132-1138. DOI: 10.1021/nl803668u.

(3) Jena, B. K.; Mishra, B. K.; Bohidar, S. Synthesis of Branched Ag Nanoflowers Based on a Bioinspired Technique: Their Surface Enhanced Raman Scattering and Antibacterial Activity. *The Journal of Physical Chemistry C* **2009**, *113* (33), 14753-14758. DOI: 10.1021/jp904689f.

(4) Verde, A.; Mangini, M.; Managò, S.; Tramontano, C.; Rea, I.; Boraschi, D.; Italiani, P.; De Luca, A. C. SERS Sensing of Bacterial Endotoxin on Gold Nanoparticles. *Frontiers in immunology* **2021**, *12*, 758410-758410. DOI: 10.3389/fimmu.2021.758410 PubMed.

(5) Dey, P. Aiming for Maximized and Reproducible Enhancements in the Obstacle Race of SERS. *ACS Measurement Science Au* **2023**, *3* (6), 434-443. DOI: 10.1021/acsmeasuresciau.3c00037.
